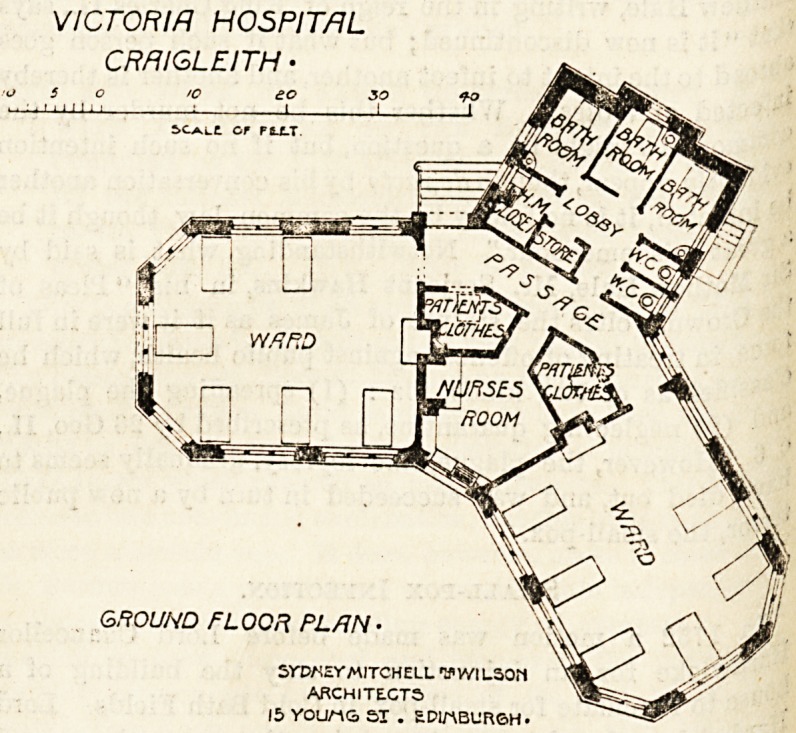# New Pavilions at the Victoria Hospital for Consumption, Craigleith, Edinburgh

**Published:** 1904-05-28

**Authors:** 


### NEW PAVILIONS AT THE VICTORIA HOSPITAL FOR CONSUMPTION, CRAIGLEITH, EDINBURGH.

Formerly this sanatorium contained about fifty beds, and lately three new pavilions have been added to it. Of these two contain twelve beds in two wards of six beds each, and one contains eight beds in two wards of four beds each. The pavilions are entirely separated from each other, and each is built in the shape of the letter Y, the arms of the latter containing the wards and, of course, facing the south, while the upright part of the letter contains the nurses' room and offices, and runs northwards.

The wards are 29 feet long, 23 feet wide, and 12 feet high, which gives nearly 1,400 cubic feet per bed; but mere cubic feet is not a matter of any great importance in a ward where windows and doors are never closed, and where cross ventilation has been attended to, and where the window space is ample.

The decision to make each unit of the sanatorium small, was undoubtedly a good one; indeed, we hold this view so strongly that we should like to have seen the units even smaller than they are, and more like the single-bedded huts found at some sanatoria—we believe at Craigleith, among others. It is more than probable that some form of hut will be the development of the future for the treatment of phthisis, and the parent buildings will become little more than administrative centres. But where pavilions have to be constructed, much might be said in favour of the form which these have taken. The details have been carefully thought out. They are only one story high. The floors are carried on brick piers and iron girders, which plan prevents dampness and ensures free circulation of air. Brick is the material used in the construction of the walls, and the walls have an air space of 2½ inches between an outer wall of 9 inches, and an inner one of 4½ inches. Inside the walls of the wards are lined with Keen's cement, those of the bath-rooms with tiles. The floors are of concrete and steel joists, and a coating of asphalt has been placed between the concrete and the wood. In all cases the angles have been rounded off.

The cost of the three pavilions was about £7,000, and of this sum Mr. W. Younger, M.P., of Moffat, presented £1,000.

We understand that the committee of management are aiming at having 100 beds available for the treatment of consumptives, and they appeal for further funds to enable them to erect five additional pavilions. Of the 32 beds just added, it is intended that eight will be reserved for patients able to pay 21s. a week each, but the others are free.

The architects were Messrs. Mitchell and Wilson, of Edinburgh.

## Figures and Tables

**Figure f1:**